# Aggressive intravenous hydration protocol of Lactated Ringer’s solution benefits patients with mild acute pancreatitis: A meta-analysis of 5 randomized controlled trials

**DOI:** 10.3389/fmed.2022.966824

**Published:** 2022-09-08

**Authors:** Fei Wu, Dong She, Qin Ao, Shan Zhang, Jin Li

**Affiliations:** Department of Emergency, The Third Affiliated Hospital of Chongqing Medical University, Chongqing, China

**Keywords:** acute pancreatitis, intravenous fluid resuscitation, aggressive intravenous hydration, Lactated Ringer, meta-analysis

## Abstract

**Objective:**

The aim of this meta-analysis was to determine the role of an aggressive intravenous hydration protocol of Lactated Ringer’s solution in patients with mild acute pancreatitis (MAP).

**Methods:**

A systematic search was conducted in PubMed, EMBASE, Cochrane Library, and China National Knowledge Infrastructure (CNKI) to identify randomized controlled trials (RCTs) published before August 19, 2022. The clinical outcomes were evaluated using the standard mean difference (SMD), mean difference (MD), risk ratio (RR), and 95% confidence interval (CI). The primary outcome was clinical improvement, while the secondary outcomes were the development of systemic inflammatory response syndrome (SIRS) and multiple organ dysfunction syndrome (MODS), relief of epigastric abdominal pain, and length of hospital stay (LoH). Statistical analysis was performed with RevMan 5.4. Grades of Recommendation, Assessment, Development, and Evaluation (GRADE) Working Group system was used to determine the quality of evidences.

**Results:**

There were five RCTs with 370 MAP patients included, and the overall methodological quality was moderate. Aggressive hydration protocol was comparable to standard hydration protocol in terms of clinical improvement (RR = 1.33, 95%CI = 0.95–1.87, *P* = 0.10; very low evidence). Fewer events of SIRS (RR = 0.48, 95%CI = 0.31–0.72, *P* < 0.001; low evidence) and MODS (RR = 0.34, 95%CI = 0.13–0.91, *P* = 0.03; moderate evidence) were reported in patients receiving aggressive hydration protocol. Meanwhile, aggressive hydration protocol also significantly relieved epigastric abdominal pain (SMD = −0.53, 95%CI = −0.81 to −0.25, *P* < 0.001; low evidence) and shorten the LoH (MD = −2.36, 95%CI = −3.17 to −1.55, *P* < 0.001; low evidence) compared with standard hydration protocol.

**Conclusion:**

For patients with MAP, aggressive hydration protocol may be more effective than standard hydration protocol at lowering SIRS and MODS rates, relieving epigastric abdominal pain, and shortening the LoH. Due to the small number of studies that are eligible and poor methodological quality of eligible studies, further studies are required to validate our findings.

## Introduction

Acute pancreatitis (AP) is an inflammation of the pancreas that typically manifests as sudden onset of epigastric abdominal pain, nausea, and epigastric tenderness to palpation (1). AP has also been one of the most common causes of gastrointestinal-related hospitalization (2). Issued data showed that approximately 30 were confirmed to have AP out from per 100,000 persons annually (3), with an increasing trend in the incidence (4). There are three subtypes of AP are classified based in severity: mild, moderately severe, and severe (5). Although the majority of patients with AP were in mild stage (6, 7), if local inflammation is not effectively treated, patients could develop local complications or even progress to systemic inflammation (8). More importantly, uncontrolled systemic inflammation is a major role in the emergence of organ failure, a condition that was previously associated with a higher risk of mortality (9). Unfortunately, there were currently no approved pharmaceutical treatments that could change how AP developed (10).

Findings from experimental studies showed that AP impairs splanchnic perfusion and the pancreatic microcirculation (11). As a result of the potential loss of the fluid in the third space, fluid resuscitation was suggested for the maintenance of hemodynamics in patients with AP (10, 12). Studies have also shown that fluid resuscitation is beneficial for lowering mortality and the occurrence of complications such as pancreatic necrosis and organ failure (10). For fluid resuscitation in AP, crystalloid solutions are preferred over colloid solutions among the available solutions (13). Numerous studies have investigated the efficacy and safety of Lactated Ringer’s (LR’s) solution as fluid resuscitation solutions, and meta-analyses recently suggested that LR’s solution might be superior to normal saline solution for the management of AP (14–16).

Traditional recommendations for fluid resuscitation varied (10, 17), but some urgently proposed concerns about aggressive hydration have been made (18). A recent meta-analysis (19) also suggested that early aggressive intravenous fluid therapy may increase the risk of pulmonary edema and acute kidney injury while not improving mortality. It should be noted that although patients of any severity and different solutions were simultaneously considered in this meta-analysis, no subgroup analysis was designed to explore how these factors affected the pooled results. It is unknown whether patients with mild AP (MAP) would benefit from an aggressive hydration protocol. Therefore, we perform this meta-analysis to determine the role of aggressive hydration of LR’s solution in the management of MAP patients.

## Materials and methods

The present meta-analysis was carried out based on the Preferred Reporting Items for Systematic Reviews and Meta-Analysis (PRISMA) statement (20). No institutional ethical approval and patients’ informed consent was necessary because this was a meta-analysis of previous studies. In addition, we did not previously register the formal protocol of our meta-analysis; however, we strictly followed the Cochrane handbook for systematic reviews of interventions (21).

### Literature identification

Two independent authors (FW and DS) searched four electronic databases, including PubMed, EMBASE, and the Cochrane library, and China National Knowledge Infrastructure (CNKI) databases, for relevant studies published before December 31, 2021. The latest search was updated on August 19, 2021. Medical subject headings terms and text words were used for development of the search strategy with the Boolean operators. The third senior author was brought into the conversation to help resolve any disagreements between the two authors. Additionally, the reference lists of the included studies were also manually screened to find additional studies. Details of search strategies for target databases are documented in Supplementary Table 1.

### Study selection

Based on the study titles, abstracts, and full texts, two independent authors (FW and QA) assessed the eligibility of the studies. Studies were included if they met the following criteria: (a) randomized controlled trials (RCTs), (b) studies of adult patients confirmed with MAP based on revised Atlanta classification (5); (c) patients in aggressive hydration group received LR’s solution 15–20 ml/kg bolus followed by infusion at 3 ml/kg/h, regardless of supplementary treatments such as probiotics; (d) patients in standard hydration group received LR’s solution 10 ml/kg bolus followed by infusion at 1.5 ml/kg/h; (e) studies reporting at least one of clinical improvement, the development of systemic inflammatory response syndrome (SIRS), the development of multiple organ dysfunction syndrome (MODS), the relief of abdominal pain, and the length of hospital stay (LoH).

According to the following criteria, we excluded ineligible studies: (a) studies that investigated fluid hydration in AP after ERCP, pediatric patient populations, animal studies, and cell lines were disregarded; (b) studies did not provide enough raw data that could be used to calculate effect estimates; (c) duplicate studies, abstracts, case reports, narrative reviews, or commentary. In case of any disagreements between the two investigators, the third investigator was consulted.

### Data extraction

Data from the included studies, including first author’s name, publication year, country, sample size, the proportion of male patients, age, and clinical outcomes, were extracted by two independent investigators (FW and SZ). The third author was consulted if there were any disagreements between the two authors. If the outcome was presented as a median with a range, we calculated the corresponding mean and standard deviation using a recognized formula (22).

### Outcomes of interesting

The primary outcome was clinical improvement within 36 h. Clinical improvement was deemed to have occurred if all of the following criteria were satisfied, including a decrease in hematocrit, BUN, and serum creatinine from baseline, a reduction in epigastric abdominal pain as measured by the visual analog scale and oral intake tolerance. We defined the development of SIRS (defined as meeting at least two of the following four criteria: heart rate > 90/min, white blood count > 12,000 or < 4,000 cells/mm^3^; respiratory rate > 20/min, partial pressure of carbon dioxide < 32 mmHg on room air, or T > 38°C or < 36°C), the development of MODS, the relief of epigastric abdominal pain [measured by visual analog scale (VAS) or numerical rating scale (NRS)], and the LoH as the secondary outcomes.

### Risk of bias assessment and the quality of evidence

Using a tool developed by the Cochrane Collaboration’s tool (23), the included studies’ quality was evaluated using seven items: random sequence generation, allocation concealment, blinding of participants and personnel, blinding of outcome assessment, incomplete outcome data, selective reporting, and other risk. Each bias item would be labeled with “high,” “unclear,” or “low” risk according to the assessment criteria. In case of any disagreements between the two investigators (FW and JL), the third investigator was consulted (DS). Moreover, we determined the quality of evidence using Grades of Recommendation, Assessment, Development, and Evaluation (GRADE) Working Group system (24).

### Statistical analysis

We utilized Review Manager version 5.4 (the Nordic Cochrane Centre, the Cochrane Collaboration, Copenhagen, 2014) to analyze the data. Standard mean difference (SMD) with the corresponding 95% confidence interval (CI) was used to express the estimate of epigastric abdominal pain because two different instruments were used for the pain assessment. On the contrary, mean difference (MD) with 95% CI was used to express the estimate of LoH. Risk ratio (RR) with 95% CI was used for expressing the estimates of dichotomous variables. Statistical heterogeneity was quantified using I2 statistic (25). Significant statistical heterogeneity was determined if the I2 value was ≥ 50%, and therefore, a random-effects model was used for meta-analysis. Otherwise, a fixed-effects model was selected for meta-analysis. Meanwhile, we compared the pooled result of the fixed-effects model and that of the random-effects model for sensitivity analysis if substantial statistical heterogeneity was detected. Publication bias test was not reported because the number of eligible studies was less than 10 (26). If *p* < 0.05, the result of statistics was significant.

## Results

### Literature retrieval

**FIGURE 1:**
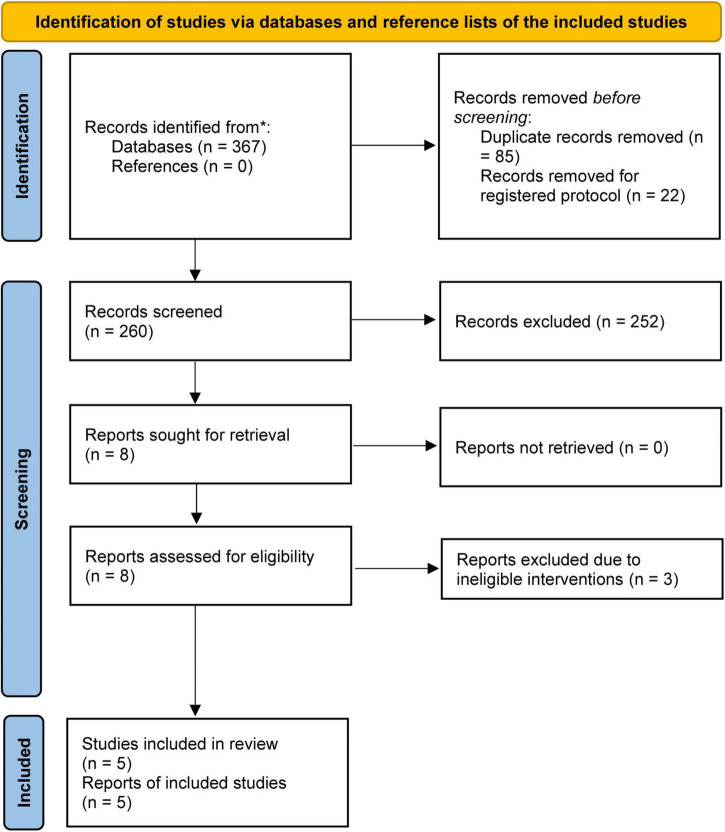
PRISMA flow diagram of study selection.

The initial literature search yielded a total of 367 relevant studies, of which 85 duplicate studies and 22 registered study protocols were removed. After checking the titles and abstracts of the remaining studies, 8 articles were retained for assessment based on full-text screening. After removing 3 studies because they used colloid solution for fluid resuscitation (27–29), five studies (30–34) were included for statistical analysis. The process of study selection is displayed in Figure 1.

### Characteristics of studies

A total of 5 studies published between 2017 and 2021 were included in this meta-analysis. Three studies (31, 32, 34) published in China, and the remaining two studies published in Thailand (33) and United States (30), respectively. The sample size of individual study ranged from 44 to 104, with a total number of 370. Four studies (30–33) used 20 ml/kg bolus in aggressive hydration group, but one study (31) used 15 ml/kg bolus as aggressive hydration protocol. In addition, one study (32) supplemented probiotics in the aggressive hydration group. Other characteristics of the included studies were summarized in Table 1.

**TABLE 1:** Basic characteristic of the included studies (*n* = 5).

Study	Origin	Detailed interventions	Etiology	Comorbidities	Sample size		Male patients		Age, years		Outcomes	Follow-up time
									
					AP	SP	AP	SP	AP	SP		
Angsubhakorn et al. (33)	Thailand	Patients in the SP group received LR’s solution 10 ml/kg bolus then 1.5 ml/kg/h; however, patients in the AP group received LR’s solution 20 ml/kg bolus then 3 ml/kg/h.	Gallstone (21), alcohol (14), hypertriglyceridemia (3), others (6)	Hypertension (19), diabetic mellitus (15), dyslipidemia (24)	22	22	18	16	46.4 ± 15.0	45.0 ± 15.1	CI, SIRS, AP	36 h
Buxbaum et al. (30)	United States	Patients in the AP group received a 20 ml/kg bolus followed by infusion at 3 ml/kg/h, and patients in SP group received a 10 ml/kg bolus followed by infusion at 1.5 ml/kg/h.	Gallstone and alcohol (48), and others (12)	Hypertension (6), diabetic mellitus (4), hyperlipidemia (1), CAD (1), COPD (1), and HIV (1)	27	33	21	24	44.4 ± 13.7	45.3 ± 12.3	CI, SIRS, AP	36 h
Wang et al. (32)	China	Patients in the AP group received a 20 ml/kg bolus at 250–500 ml/h, followed by infusion at 3 ml/kg/h; however, patients in SP group received a 10 ml/kg bolus at 250–500 ml/h, followed by infusion at 1.5 ml/kg/h.	Gallstone (32), alcohol (19), hypertriglyceridemia (31), others (22)	n.a.	52	52	29	28	42.81 ± 1.26	42.04 ± 1.15	SIRS, MODS, LoH	36 h
Li et al. (31)	China	Patients in the AP group received a 20 ml/kg bolus followed by infusion at 3 ml/kg/h, and patients in SP group received a 10 ml/kg bolus followed by infusion at 1.5 ml/kg/h.	Gallstone (50), alcohol (15), hypertriglyceridemia (24), others (11)	n.a.	50	50	36	27	44.3 ± 10.3	46.2 ± 12.5	CI, SIRS, MODS, AP, LoH	36 h
Liu et al. (34)	China	Patients in the AP group received a 15 ml/kg bolus, and patients in SP group received a 10 ml/kg bolus.	n.a.	n.a.	35	27	n.a.	n.a.	45.31 ± 13.49	46.90 ± 11.79	SIRS, MODS, LoH	36 h

LR, lactated ringer; AP, aggressive hydration protocol; SP, standard hydration protocol; CAD, coronary artery disease; COPD, chronic obstructive pulmonary disease; HIV, human immunodeficiency virus; CI, clinical improvement; SIRS, systemic inflammatory response syndrome; MODS, multiple organ dysfunction syndrome; AP, abdominal pain; LoH, length of hospitalization; n.a., not applicable.

### Risk of bias

**FIGURE 2:**
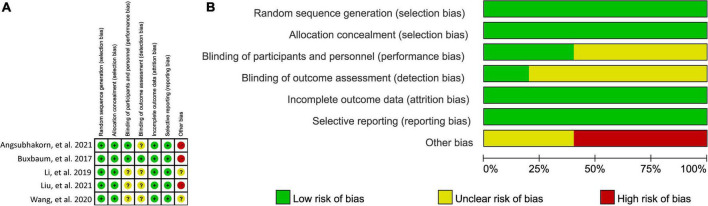
Risk of bias summary **(A)** and graph **(B)**. “green (+),” “yellow (?),” and “red (−),” indicates “low,” “unclear,” and “high” risk of bias, respectively.

Details of risk of bias assessment of all eligible studies were displayed in Figure 2. All studies (30–34) correctly generated and concealed random sequence and therefore were labeled as “low” risk at selection bias domain. Two studies (30, 33) reported to blind participants and personnel, and therefore labeled as “low” risk at performance bias. Detection bias was considered as “low” risk in only one study (30). Incomplete outcome data and selective reporting data were considered as “low” risk in all eligible studies (30–34). Three studies (30, 33, 34) were labeled as “high” risk in other risk domain due to small sample size.

### Clinical improvement

**FIGURE 3:**
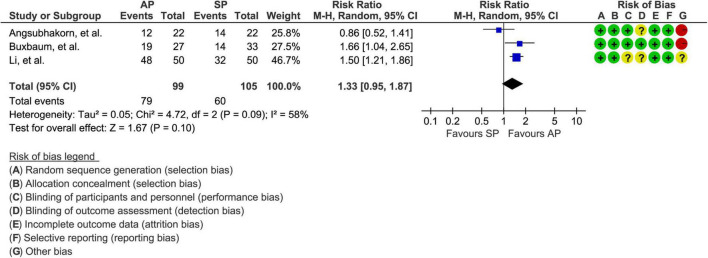
Forest plot of clinical improvement. AP, aggressive hydration protocol; SP, standard hydration protocol; CI, confidence interval.

A total of 3 studies involving 204 patients (30, 31, 33) reported the data of clinical improvement. The random-effects model was used because there was significant heterogeneity across studies (*P* = 0.09, I2 = 58%). As shown in Figure 3, the pooled results showed no statistically significant difference in clinical improvement between aggressive hydration protocol and standard hydration protocol (79.8% vs. 57.1%; RR = 1.33; 95% CI = 0.85–1.87; *P* = 0.10), which was only supported by the very low evidence (Table 2).

**TABLE 2:** Summary of findings based on GRADE system.

Certainty assessment							No of patients		Effect		Certainty	Importance
No of studies	Study design	Risk of bias	Inconsistency	Indirectness	Imprecision	Other considerations	AP	SP	Relative (95% CI)	Absolute (95% CI)		
**Clinical improvement**												
3	Randomized trials	Serious^a^	Serious^b^	Not serious	Serious^c^	None	79/99 (79.8%)	60/105 (57.1%)	**RR 1.33** (0.95–1.87)	**189 more per 1,000** (from 29 fewer to 497 more)	⨁◯◯◯ Very low	CRITICAL
**SIRS**												
5	Randomized trials	Serious^d^	Serious^e^	Not serious	Not serious	None	23/178 (12.9%)	52/192 (27.1%)	**RR 0.48** (0.31–0.72)	**141 fewer per 1,000** (from 187 fewer to 76 fewer)	⨁⨁◯◯ Low	CRITICAL
**MODS**												
3	Randomized trials	Serious*^f^*	Not serious	Not serious	Not serious	None	5/137 (3.6%)	14/129 (10.9%)	**RR 0.34** (0.13–0.91)	**72 fewer per 1,000** (from 94 fewer to 10 fewer)	⨁⨁⨁◯ Moderate	CRITICAL
**Epigastric abdominal pain**												
3	Randomized trials	Serious^a^	Not serious	Not serious	Serious^c^	None	99	105	−	SMD **0.53 SD lower** (0.81 lower to 0.25 lower)	⨁⨁◯◯ Low	CRITICAL
**LoH**												
3	Randomized trials	Serious*^f^*	Not serious	Not serious	Serious^c^	None	137	129	−	MD **2.36 lower** (3.17 lower to 1.55 higher)	⨁⨁◯◯ Low	CRITICAL

AP, aggressive hydration protocol; SP, standard hydration protocol; SIRS, systemic inflammatory response syndrome; MODS, multiple organ dysfunction syndrome; LoH, length of hospitalization; CI: confidence interval; SMD, standard mean difference; MD: mean difference; RR: risk ratio.

^a^Two eligible studies were rated as having high risk.

^b^Heterogeneity examination reported an inconsistency factor of 58%.

^c^Three studies were rated as having high risk.

^d^One study was rated as having high risk.

^e^Only 99 patients were accumulated in aggressive hydration group.

^f^One study was rated as having high risk.

### Systemic inflammatory response syndrome

**FIGURE 4:**
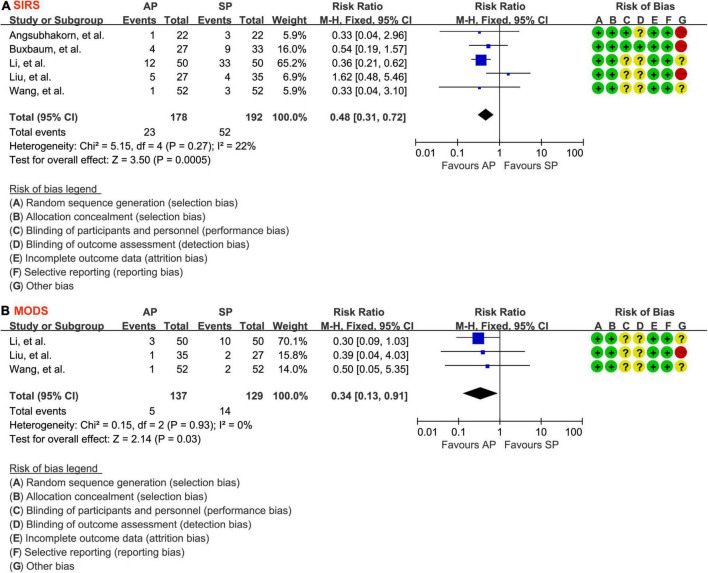
Forest plot of the development of SIRS **(A)** and MODS **(B)**. SIRS, systemic inflammatory response syndrome; MODS, multiple organ dysfunction syndrome; AP, aggressive hydration protocol; SP, standard hydration protocol; CI, confidence interval; M-H, Mantel-Haenszel.

The events of SIRS were reported in all eligible studies (30–34) involving 370 patients. Because there was no evidence of statistical heterogeneity between studies (*P* = 0.27, I2 = 22%), the fixed-effects model was therefore used in meta-analysis. As shown in Figure 4A, compared with standard hydration protocol, fewer patients suffered from SIRS in the aggressive hydration protocol group (12.9% vs. 27.1%; RR = 0.48; 95% CI = 0.31–0.72; *P* < 0.001), which was supported by the low evidence (Table 2).

### Multiple organ dysfunction syndrome

The events of MODS were reported in 3 studies (31, 32, 34) involving 266 patients. The fixed-effects model was chosen because significant statistical heterogeneity between studies was not detected (*P* = 0.93, I2 = 0.0%), so a fixed-effect model was selected. As shown in Figure 4B, aggressive hydration protocol was associated with lower incidence of MODS compared with standard hydration protocol (3.6% vs. 10.9%; RR = 0.34; 95% CI = 0.13–0.91; *P* = 0.03), which was supported by the moderate evidence (Table 2).

### Epigastric abdominal pain

**FIGURE 5:**
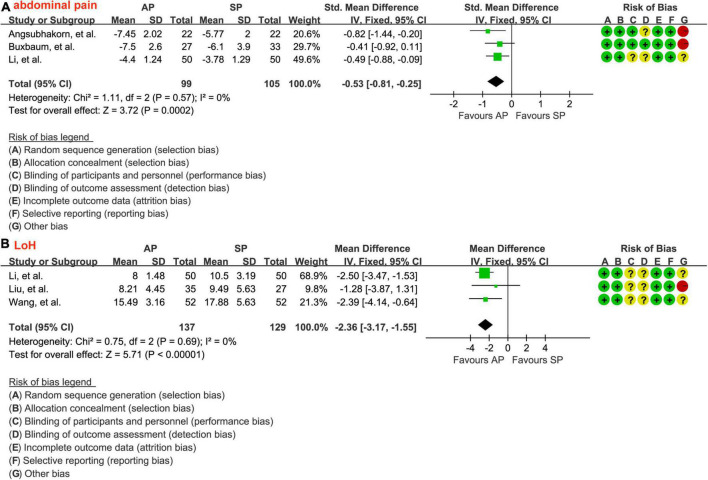
Forest plot of the relief of epigastric abdominal pain **(A)** and the LoH **(B)**. LoH, length of hospital stay; AP, aggressive hydration protocol; SP, standard hydration protocol; SD, standard deviation; CI, confidence interval; IV, inverse variance.

A total of 3 studies (30, 31, 33) involving 204 patients reported the data of the relief of epigastric abdominal pain and a fixed-effects model was selected because there is insignificant heterogeneity between studies (*P* = 0.57, I2 = 0.0%). As shown in Figure 5A, aggressive hydration protocol significantly relieved the epigastric abdominal pain compared with standard hydration protocol (SMD = −0.53; 95% CI = −0.81 to −0.25; *P* < 0.001), which was supported by the low evidence (Table 2).

### Length of hospital stay

The data of LoH after treatment were reported by a total of 3 studies involving 266 patients, and there was no significant heterogeneity across studies (*P* = 0.69, I2 = 0.0%). The result from the fixed-effects model suggested that, as shown in Figure 5B, patients who received aggressive hydration protocol had shorter LoH than those patients who received standard hydration protocol (MD = −2.36; 95% CI = −3.17 to −1.55; *P* < 0.001), which was supported by the low evidence (Table 2).

## Discussion

### Main findings

Although a previous meta-analysis suggested that aggressive hydration protocol did not reduce mortality and might increase the risk for acute kidney injury and pulmonary edema; however, the benefit of aggressive hydration protocol has not yet been determined in patients with MAP. This is the first meta-analysis, to the best of our knowledge, which evaluated the efficacy and safety of aggressive intravenous hydration protocol of LR’s solution in patients with MAP. Very low evidence suggested a comparable clinical improvement between aggressive hydration protocol and standard protocol of LR’s solution. However, low to moderate evidences suggested that aggressive hydration protocol of LR’s solution also significantly decreased the risk of developing SIRS and MODS, accelerated the relief of epigastric abdominal pain, and shorten the LoH among patients with MAP.

### Comparison with previous meta-analyses

One meta-analysis (19) has been done to date to determine whether aggressive intravenous fluid therapy helps patients with AP have fewer mortality and better clinical outcomes. The authors of this meta-analysis, which included 11 eligible studies (4 RCTs and 7 cohort studies), came to the conclusion that early aggressive intravenous fluid therapy did not reduce mortality but might increase the risk of acute kidney injury and pulmonary edema. It should be noted that patients of any severity were not separately analyzed in this meta-analysis, which simultaneously included studies with diverse designs into individual analysis. As a result, previous meta-analysis could not generate a firm conclusion for a specific population. We significantly improve the homogeneity of studies when compared to previous meta-analysis by only including patients who were diagnosed with MAP and then received an aggressive hydration protocol of LR’s solution. Additionally, this meta-analysis only included RCTs with full texts, greatly increasing the reliability of conclusions.

### Strengths and limitations

There were some advantages to the present meta-analysis. First, this meta-analysis included the most comprehensive RCTs on the “head-to-head” comparison of aggressive hydration protocol and standard hydration protocol in the management of patients with mild AP. Second, we were able to better capture all potentially eligible studies because there were no restrictions on publication language or status in this meta-analysis. Third, clinical practitioners may use the strong evidence from this meta-analysis to guide their selection of fluid resuscitation strategies.

This meta-analysis had some limitations. First, only a small number of eligible studies with small sample sizes were included in this meta-analysis, which could have weakened the validity of all pooled results. Second, because there were so few eligible studies included, the publication bias test was not conducted. As a result, we were unable to eliminate the negative impact of publication bias on all pooled results. Third, due to sparse data, this meta-analysis did not evaluate laboratory parameters such as serum creatinine, inflammatory markers, and procalcitonin level. Forth, most eligible studies (80%) did not clearly describe details of blinding of participants, personnel, and outcome assessment. Therefore, RCTs with high-quality and adequate sample size are still urgently needed to confirm our findings. Fifth, the aggressive protocol was defined differently in one study (31) than it was in the other, and probiotics were added in the aggressive hydration group in one study (32). The variations may therefore introduce bias into the pooled results. Sixth, mortality was not a reported outcome in all studies. Future studies should take this outcome into account when comparing the efficacy and safety of different hydration protocols. Seventh, because most eligible studies were conducted in China, caution should be used when interpreting the applicability of our findings. Finally, although we conducted this meta-analysis in strict accordance with the Cochrane handbook for systematic reviews of interventions, we did not register the formal protocol on any public platform, which may introduce potential bias to impair the reliability of the pooled results.

## Conclusion

In contrast to standard hydration protocol, aggressive hydration protocol may effectively reduce the rates of SIRS and MODS, relieve epigastric abdominal pain, and shorten the LoH among patients with MAP more efficaciously than standard hydration protocol. However, given the small number of eligible studies, inadequate sample sizes, and poor overall methodological quality that were included in this meta-analysis, more studies are required to validate our findings.

## Data availability statement

The original contributions presented in this study are included in the article/Supplementary material, further inquiries can be directed to the corresponding author/s.

## Author contributions

FW and DS: conceptualization, methodology, writing original draft preparation, and formal analysis. FW: software and visualization. QA and SZ: validation. FW and JL: investigation and resources. DS and JL: data curation and writing review and editing. DS: supervision, project administration, and funding acquisition. All authors have read and agreed to the published version of the manuscript.

## Supplementary material

Supplementary Table 1 Search strategy.
