# Supplementary material for: Aggressive intravenous hydration protocol of Lactated Ringer’s solution benefits patients with mild acute pancreatitis: A meta-analysis of 5 randomized controlled trials
Source: Front Med (Lausanne). 2022 Sep 8;9:966824. doi: 10.3389/fmed.2022.966824 (PMC9492986; doi:10.3389/fmed.2022.966824)
Supplement: Supplementary Table 1 — Search strategy. [file Data_Sheet_1.pdf]

Search strategy of PubMed

....

No.	Search Details	Results
7	("Pancreatitis"[MeSH Terms] OR (((("Acute"[All Fields] OR "acutely"[All Fields] OR "acutes"[All Fields]) AND ("Edema"[MeSH Terms] OR "Edema"[All Fields] OR "Edematous"[All Fields] OR "oedematous"[All Fields])) AND "Pancreatitides"[Title/Abstract]) OR "acute edematous pancreatitis"[Title/Abstract] OR "pancreatic parenchymal edema"[Title/Abstract] OR "pancreatic parenchymal edema"[Title/Abstract] OR "acute pancreatitis"[Title/Abstract] OR "acute pancreatitides"[Title/Abstract] OR "mild acute pancreatitis"[Title/Abstract])) AND ("Ringer's Lactate"[MeSH Terms] OR ("hartmanns solution"[Title/Abstract] OR "lactated ringers solution"[Title/Abstract] OR "ringers lactate"[Title/Abstract] OR "lactated ringer s"[Title/Abstract] OR "lactated ringer s solution"[Title/Abstract] OR "lactated ringer solution"[Title/Abstract] OR "hartmann s solution"[Title/Abstract]))	87
6	"Ringer's Lactate"[MeSH Terms] OR "hartmanns solution"[Title/Abstract] OR "lactated ringers solution"[Title/Abstract] OR "ringers lactate"[Title/Abstract] OR "lactated ringer’s"[Title/Abstract] OR "lactated ringer’s solution"[Title/Abstract] OR "lactated ringer solution"[Title/Abstract] OR "hartmann’s solution"[Title/Abstract]	3,589
5	"hartmanns solution"[Title/Abstract] OR "lactated ringers solution"[Title/Abstract] OR "ringers lactate"[Title/Abstract] OR "lactated ringer’s"[Title/Abstract] OR "lactated ringer’s solution"[Title/Abstract] OR "lactated ringer solution"[Title/Abstract] OR "hartmann’s solution"[Title/Abstract]	2,791
4	"Ringer's Lactate"[MeSH Terms]	1,493
3	"Pancreatitis"[MeSH Terms] OR (((("Acute"[All Fields] OR "acutely"[All Fields] OR "acutes"[All Fields]) AND ("Edema"[MeSH Terms] OR "Edema"[All Fields] OR "Edematous"[All Fields] OR "oedematous"[All Fields])) AND "Pancreatitides"[Title/Abstract]) OR "acute edematous pancreatitis"[Title/Abstract] OR "pancreatic parenchymal edema"[Title/Abstract] OR "pancreatic parenchymal edema"[Title/Abstract] OR "acute pancreatitis"[Title/Abstract] OR "acute pancreatitides"[Title/Abstract] OR "mild acute pancreatitis"[Title/Abstract])	60,457
2	((("Acute"[All Fields] OR "acutely"[All Fields] OR "acutes"[All Fields]) AND ("Edema"[MeSH Terms] OR "Edema"[All Fields] OR "Edematous"[All Fields] OR "oedematous"[All Fields])) AND "Pancreatitides"[Title/Abstract]) OR "acute edematous pancreatitis"[Title/Abstract] OR "pancreatic parenchymal edema"[Title/Abstract] OR "pancreatic parenchymal	26,413

	edema"[Title/Abstract] OR "acute pancreatitis"[Title/Abstract] OR "acute pancreatitides"[Title/Abstract] OR "mild acute pancreatitis"[Title/Abstract]	
1	"Pancreatitis"[MeSH Terms]	55,196

....

Search strategy of EMBASE

……………………………………………….

No.	Query	Results
#7	#3 AND #6	208
#6	#4 OR #5	9053
#5	'ringer lactate solution'/exp	8814
#4	'ringers lactate':ti,ab,kw OR 'lactated ringers solution':ti,ab,kw OR 'lactated ringer solution':ti,ab,kw OR 'hartmanns solution':ti,ab,kw	549
#3	#1 OR #2	47546
#2	'acute pancreatitis'/exp	35172
#1	'acute edematous pancreatitides':ti,ab,kw OR 'pancreatic parenchymal edema':ti,ab,kw OR 'acute pancreatitis':ti,ab,kw OR 'acute pancreatitides':ti,ab,kw OR 'mild acute pancreatitis':ti,ab,kw	39762

……………………………………………….

Search strategy of Cochrane library

....

ID	Search	Hits
#1	(Acute Edematous Pancreatitides):ti,ab,kw OR (Acute Edematous Pancreatitis):ti,ab,kw OR (Acute Edematous Pancreatitis):ti,ab,kw OR (Pancreatic Parenchymal Edema):ti,ab,kw OR (Acute Pancreatitis):ti,ab,kw	2625
#2	(Acute Pancreatitides):ti,ab,kw OR (mild acute pancreatitis):ti,ab,kw	287
#3	#1 or #2	2625
#4	MeSH descriptor: [Pancreatitis] explode all trees	1432
#5	#3 or #4	3209
#6	(Hartmanns Solution):ti,ab,kw OR (Lactated Ringers Solution):ti,ab,kw OR (Ringers Lactate):ti,ab,kw OR (Lactated Ringer's):ti,ab,kw OR (Lactated Ringer's Solution):ti,ab,kw	871
#7	(Lactated Ringer Solution):ti,ab,kw OR (Hartmann's Solution):ti,ab,kw	756
#8	#6 or #7	967
#9	MeSH descriptor: [Ringer's Lactate] explode all trees	267
#10	#8 or #9	1076
#11	#5 and #10	59

....

Search strategy of CNKI

检索范围（search scope）：**总库**（all databases）（主题 [subject]：胰腺炎（精确）(pancreatitis [precise])）OR（主题 [subject]：急性胰腺炎（精确）(acute pancreatitis [precise])）OR（主题 (subject)：急性水肿性胰腺炎（精确）(Acute Edematous Pancreatitides [precise])）OR（主题 (subject)：轻度急性胰腺炎（精确）(mild acute pancreatitis [precise])）OR（主题 (subject)：轻症急性胰腺炎（精确）(mild acute pancreatitis [precise])）OR（主题 (subject)：胰腺实质水肿（精确）(Pancreatic Parenchymal Edema [precise]） AND （（主题 (subject)：乳酸林格氏液（精确）(Lactated Ringers Solution [precise])）OR（主题 (subject)：乳酸钠林格液（精确）(Lactated Ringers Solution [precise])）OR（主题 (subject)：戴维森氏液（精确）(Hartmann's Solution [precise])）OR（主题 (subject)：哈特曼氏溶液（精确）(Hartmann's Solution [precise])）OR（主题 (subject)：乳酸林格（精确）(Ringers Lactate [precise])））13
